# An efficient and accurate semi-analytical matching technique for waveguide-fed antennas

**DOI:** 10.1038/s41598-024-54034-8

**Published:** 2024-02-16

**Authors:** Edoardo Negri, Walter Fuscaldo, Silvia Tofani, Paolo Burghignoli, Alessandro Galli

**Affiliations:** 1 Department of Information Engineering, Electronics and Telecommunications, Sapienza University of Rome, 00184 Rome, Italy; 2 Istituto per la Microelettronica e Microsistemi, Consiglio Nazionale delle Ricerche, 00133 Rome, Italy

**Keywords:** Electrical and electronic engineering, Electronics, photonics and device physics

## Abstract

Several RF and microwave radiating devices, such as horn antennas, Fabry–Perot cavity antennas, and aperture-fed focusing devices, are excited through rectangular waveguides. The impedance matching of the overall system (from the waveguide feed to the radiating aperture) is a task of crucial importance that is often addressed by means of brute-force parameter-sweep full-wave analyses or blind optimization algorithms. In both cases, a significant amount of memory and time resources are required. For this purpose, we propose here a simple, yet effective solution, which only requires a single full-wave simulation and a semi-analytical procedure. The former is used to retrieve the antenna input impedance at the end of the waveguide port excitation. The semi-analytical procedure consists in a transmission-line equivalent circuit that models two waveguide discontinuities (namely two capacitive irises) within the waveguide section, whose position and geometric features are finely tuned to obtain a satisfactory impedance matching around the working frequency. The proposed method is shown to be effective in diverse and attractive application-oriented contexts, from the impedance matching of a Fabry–Perot cavity antenna to that of a wireless near-field link between two aperture-fed focusing devices. A remarkable agreement between full-wave simulations and numerical results is found in all cases. Thanks to its versatility, simplicity, and a rather low demand of computational resources, the proposed approach may become an essential tool for the effective design of waveguide-fed antennas.

## Introduction

Rectangular waveguides (RWGs) are a common form of feeders used at microwave^1^, millimeter-wave^2^ and terahertz (THz) frequencies^3^ due to their simplicity of manufacture, relatively high level of power handling, and frequency-scaling feature. They are commonly used to excite horn antennas^4^ but also quasi-resonant slots etched on the ground plane of planar antennas in order to provide a horizontal magnetic dipole (HMD) source^2^. In particular, an HMD has been used to excite different kinds of leaky-wave antennas (LWAs): Fabry–Perot cavity (FPC) LWAs^2^, planar two-dimensional (2-D) LWAs^3,5^, 2-D periodic LWAs^6^, and, recently, resonant Bessel-beam launchers (BBLs)^7^. However, matching issues may appear when RWGs physically excite the slot etched on the ground plane. This is due to the considerably different transverse size of the feeder and the LWA cavity (namely, from fractions of wavelengths to tens of wavelengths, respectively) and also to the transition from a guided wave to a radiating one. A simple and effective solution to this issue is the insertion of one or more (depending on the targeted bandwidth) waveguide discontinuities capable of matching the waveguide impedance^8^. As is well known, the discontinuity generates reflected waves and a storage of reactive energy in its vicinity. The latter is due to the excitation of waveguide higher-order modes which are evanescent, i.e., decay exponentially as the distance from the discontinuity increases (because the device has been designed in order to let propagate only the fundamental mode^9^). Therefore, the excited higher-order modes act as a perturbation of the fundamental mode, giving rise to phase shifts and impedance variations near the waveguide discontinuity that could be used for matching purposes^8,9^.

One approach to optimize the design parameters of the waveguide discontinuities is to resort to parameter-sweep full-wave simulations, possibly driven by optimization algorithms. However, depending on the structure under analysis, this solution could be computationally expensive. In order to circumvent this issue, one might consider an approximate analytical description of canonical waveguide discontinuities, to be used in equivalent transmission-line models (TLMs). Indeed, when the magnitude of the higher-order modes excited by the waveguide discontinuity is small, a variational method^10^ may be used in order to describe the discontinuity with a specific reactive lumped element in an equivalent transmission-line circuit^9^.

While the characterization of waveguide discontinuities through variational methods^8–10^ and their subsequent extensions to more elaborate cases^11–13^ are well known, its application to TLMs for matching the impedance of waveguide-fed radiating devices is very little explored. Still, recent works on Fabry–Perot cavity antennas as well as near-field radiating devices make more and more use of waveguide feeds (see, e.g.,^3,5,7,14^), especially for the upper range of millimeter waves and for THz waves where coaxial cables are no longer available and waveguide transitions represent the most efficient solutions^3^. In such structures, the minimization of insertion losses is instrumental to maximize the realized gain, or, more in general, the radiated power density, and in turn the overall efficiency of the system.

For these reasons, we develop here a semi-analytical method assisted by full-wave simulations that is capable of deriving in a fast and accurate way the geometric parameters of a pair of waveguide discontinuities placed within a waveguide section in order to get a satisfactory impedance matching. More precisely, we consider a waveguide section where two capacitive irises are placed at specific locations to minimize the reflection coefficient in numerous and diverse scenarios of interest for the antenna and microwave-up-to-THz communities. A similar approach is given by the double-stub matching technique^15^ for which two reactive elements are placed at arbitrary distances from the load but the possible matched loads are limited. The main difference is that, in such a technique, reactive loads are typically obtained through two open- or short-circuited transmission lines, whereas here we use simple waveguide discontinuities (viz., capacitive diaphragms) in RWG feeders.

**Figure 1:**
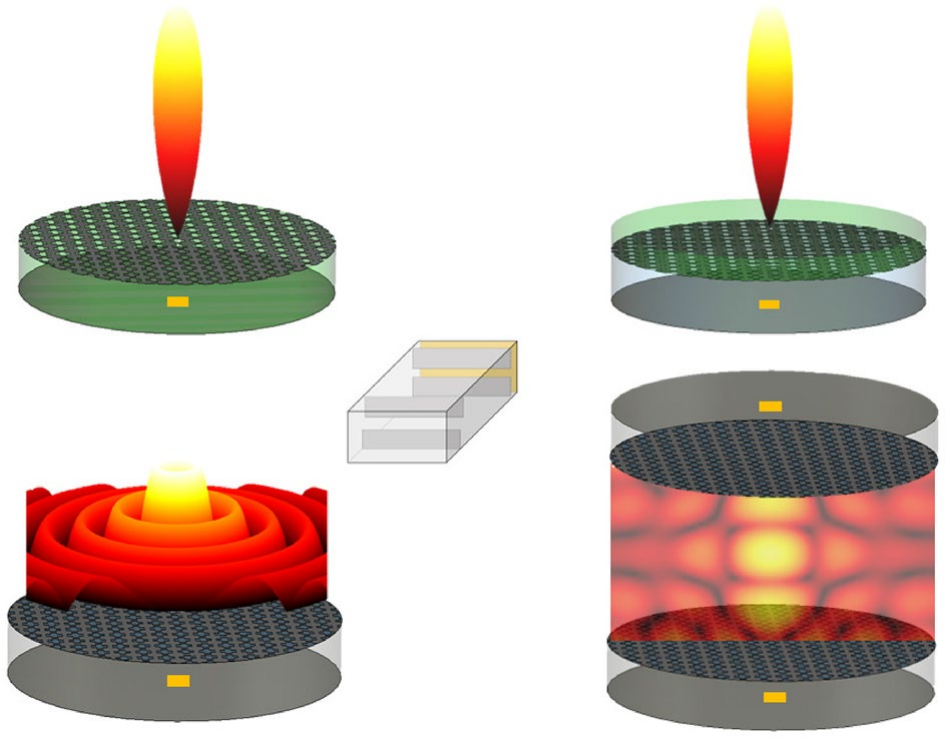
Different practical scenarios, given by electrically large radiating devices (upper part) and compact resonant BBLs (bottom part), of a RWG feeder matched through capacitive irises.

In particular, we test our proposed approach in the following four different scenarios (see Fig. 1): *i)* a canonical Fabry–Perot cavity leaky-wave antenna (FPC-LWA) based on a *thin* partially reflecting sheet (PRS) radiating a pencil beam at broadside in far field (see, Fig. 1, top-left corner)^14^; *ii)* a wideband version of an FPC-LWA based on a *thick* PRS (see, Fig. 1, top-right corner)^16^; *iii)* a *Bessel-beam launcher*, i.e., a focusing planar device radiating a Bessel beam in the radiative near-field region (see, Fig. 1, bottom-left corner)^17,18^; *iv)* two Bessel-beam launchers coupled in the radiative near-field so as to realize a wireless link (see, Fig. 1, bottom-right corner)^7^. In all the abovementioned scenarios, an excellent impedance matching is obtained, based on our method. A remarkable agreement is also observed between numerical results and full-wave simulations, thus confirming the consistency, the accuracy, and the efficiency of the proposed procedure.

The paper is organized as follows. In Section “Theoretical approach”, the approximate analytical models for the description of waveguide discontinuities in an RWG are briefly summarized. An original useful formula is also provided to have a compact and more complete analytical description. These results are exploited in Section “Numerical implementation” to develop an equivalent TLM of a waveguide section comprising two uncoupled waveguide discontinuities. The geometrical parameters of this TLM can be finely tuned to match the waveguide characteristic impedance with the input impedance of the radiating device. The latter is retrieved by means of a single full-wave simulation taking advantage of the low coupling between the radiating aperture and the waveguide feed. In Section “Case studies”, the proposed method is tested and validated in numerous and diverse scenarios. Conclusion are finally drawn in Section “Conclusion”.

## Theoretical approach

In this Section, the well-known theoretical description of the discontinuities given by the capacitive irises in a rectangular waveguide is reported along with an original, analytical derivation of the required coefficients.

### Equivalent circuit for matching irises

As previously discussed, waveguide discontinuities can typically be represented through an equivalent circuit. As is known, general variational approaches for the determination of the elements occurring in such equivalent circuits have been developed by Schwinger^10^. For common structures it is possible to characterize the discontinuity with a simple two-port network. In particular, an asymmetric electrically thin and lossless diaphragm on a parallel-plate waveguide (PPW) can be represented through a purely imaginary shunt impedance, i.e., a shunt capacitance on the equivalent transmission line of the waveguide^9^. This is due to the field distribution of the fundamental transverse electromagnetic (TEM) mode in the PPW. If the working frequency has been set in order to let propagate only the fundamental mode, the higher-order modes excited on the discontinuity are evanescent and they generate reactive electric energy. Since the electric field of the dominant mode is continuous on the diaphragm plane and the magnetic field changes due to the presence of the induced current on the metal plate, the best circuital representation of the reactive energy contribution is given by a shunt capacitance. The analytical expression for its susceptance value, exploited for the RWG case in (5) and (6), is obtained through an ad-hoc variational method which exploits higher-order vanishing modes to characterize the waveguide discontinuity^9^.

**Figure 2:**
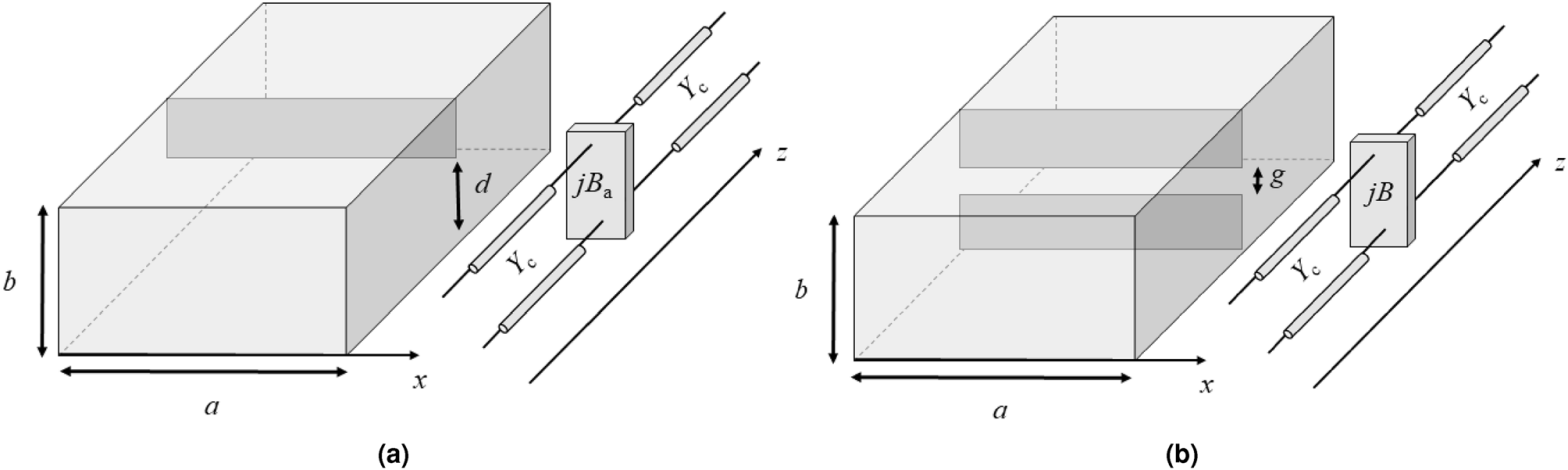
Pictorial representation of (**a**) an asymmetric capacitive iris and of (**b**) a symmetric iris in a rectangular waveguide along with their equivalent transmission-line model.

The analytical description achieved for an asymmetric diaphragm in a PPW^9^ (considering *b* the plate separation according to the geometry of Fig. 2a) can be extended in order to represent the same discontinuity (which leaves a metallic-free height *d*) in an RWG of width and height *a* and *b*, respectively (see Fig. 2a). In this case, the transverse-electric mode TE_10_ is the fundamental one. Therefore, the electric field still has only a vertical component but with a sinusoidal (and not constant as for the TEM case) distribution along the *x* direction ($\textbf{E}=E_y\mathbf {y_0}$ with ${E_y=E_0\sin (\pi x/a)}$). However, the waveguide discontinuities are along the vertical direction *y* and, then, the modal field distribution of the RWG problem satisfies the same boundary conditions of the PPW problem. The only new field contribution is the *z* component of the magnetic field which satisfies the proper boundary conditions because they are derivable from the curl of the electric field^9^. Therefore, the same equations can be considered for the susceptance $B_{\mathrm{s}}$ of an asymmetric capacitive diaphragm in a PPW and in an RWG by considering the proper propagation constant for both the fundamental and the higher-order modes. In particular, while for the PPW modes the propagation constants of the fundamental TEM and higher-order TE modes are the wavenumber $k=\omega \sqrt{\mu \varepsilon }$ (being $\omega$, $\mu$, and $\varepsilon$ the angular working frequency, the magnetic permeability of the material inside the waveguide, and its electric permittivity, respectively) and $k_{zn} = \sqrt{k^2-(n\pi /b)^2}$ (with *n* = 1, 2, ...), respectively, for the RWG the wavenumbers of the TE_1n_ modes are:

1 $$\begin{aligned} k_{zn}=\sqrt{k^2-(\pi /a)^2-(n\pi /b)^2} \end{aligned}$$

It is worth pointing out that the modes considered in the waveguide problem are *proper* modes in the sense that they satisfy the Sommerfeld radiation boundary condition, i.e., the root choice in (1) is the one for which we have $\textrm{Im}(k_{zn})<0$. Therefore, the upper and lower bounds of the normalized susceptance value of an asymmetric diaphragm in a RWG are given by the following expressions^9^:

2 $$\begin{aligned} \frac{B^\text{up}_\mathrm{a}}{Y_\mathrm{c}} = \frac{4{k_{z0}}b}{\pi }\left[ \ln \csc \frac{\pi d}{2b}+\sum _{n=1}^\infty \left( \frac{\pi }{jb{k_{zn}}}-\frac{1}{n}\right) P^2_{n0}\right] \end{aligned}$$

and

3 $$\begin{aligned} \frac{B^\text{dw}_\mathrm{a}}{Y_\mathrm{c}} = \frac{4{k_{z0}}b}{\pi }\left[ \ln \csc \frac{\pi d}{2b}+\frac{[\pi /(jbk_{z1})-1]\alpha _1^2}{1+[\pi /(jbk_{z1})-1]\alpha _2^2}\right] \end{aligned}$$

where $\alpha _1=\cos ^2\left(\frac{\pi d}{2b}\right)$, $\alpha _2=1-\alpha _1$, $P_{n0}$ are specific coefficients discussed below, and $Y_{\mathrm{c}}$ is the characteristic admittance of the fundamental TE_10_ mode in propagation, i.e., $Y_{\mathrm{c}}={k_{z0}}/(\omega \mu )$. At this point, the equivalent susceptance of the matching iris can be obtained through the arithmetical mean of its upper- and lower-bound values:

4 $$\begin{aligned} B_{\textrm{a}}=\frac{{B}_\mathrm{a}^{\textrm{up}}+{B}_\mathrm{a}^{\textrm{dw}}}{2}. \end{aligned}$$

However, for practical applications, a symmetric capacitive diaphragm, which produces an iris slot in the waveguide of height *g* (see Fig. 2b), is considered. In this case, the image theory can be applied thanks to the symmetry of the problem. Since the symmetry of the structure imposes the excitation of higher-order modes with a zero component of longitudinal electric field on the symmetry plane, a conducting plane may be placed there^9^. Therefore, the problem is reduced to the case of asymmetrical diaphragm for which *b* is replaced by *b*/2 and *d* by *g*/2 obtaining:

5 $$\begin{aligned} \frac{B^{\textrm{up}}}{Y_\mathrm{c}} = \frac{2k_{z0}b}{\pi }\left[ \ln \csc \frac{\pi g}{2d}+\sum _{n=1}^\infty \left( \frac{2\pi }{jbk_{zn}}-\frac{1}{n}\right) P^2_{n0}\right] \end{aligned}$$

and

6 $$\begin{aligned} \frac{B^{\textrm{dw}}}{Y_\mathrm{c}} = \frac{2k_{z0}b}{\pi }\left[ \ln \csc \frac{\pi g}{2b}+\frac{[2\pi /(jbk_{z1})-1]\alpha _1^2}{1+[2\pi /(jbk_{z1})-1]\alpha _2^2}\right] \end{aligned}$$

being the $P_{n0}$ coefficients given by (10) (see further below) with $\alpha _1$ and $\alpha _2$ computed by replacing *d* with *g*. In the following, the average between the upper and lower bound values, *B*, for the susceptance is chosen. Since we have always considered $g/b<0.5$, the maximum difference between the two susceptance bounds is less than 4% and, therefore, the average value is in error by less than 2%^9^. As shown in the Section related to the case studies, this minimal error does not affect the matching-technique performance.

### Analytical determination of the $P_{n0}$ terms

The $P_{n0}$ terms appearing in (5) are but the $m=0$ coefficients of a cosine expansion of the term $\cos (n\pi y/b)$ which represents higher-order eigenmodes in a RWG. As is well known, these functions can easily be related to the *n*-th powers of cosine contributions; an identity that naturally leads to the definition of Chebyshev polynomials. Therefore, by considering the natural choice of a Chebyshev polynomial-based expansion, the following relation is derived^9^:

7 $$\begin{aligned} {\sum _{m=0}^n} P_{nm}\cos (m\theta )=\cos (n\pi y/b)=T_n(\alpha _1+\alpha _2\cos \theta ) \end{aligned}$$

where in the last step we used the definition of Chebyshev polynomials $T_n(\cdot )$ of order *n* and the relation $\cos (\pi y/b) = \alpha _1+\alpha _2\cos \theta$.

At this stage, it is convenient to express the Chebyshev polynomial in explicit form:

8 $$\begin{aligned} {\sum _{m=0}^n}P_{nm}\cos (m\theta )=\sum _{p=0}^nT_{np}\sum _{q=0}^p{p\atopwithdelims ()q}\alpha _1^{p-q}\alpha _2^q\cos ^q\theta \end{aligned}$$

where we used the Newton binomial expansion to express the *p*-th power of the argument of $T_n(\cdot )$ given by $\alpha _1+\alpha _2\cos \theta$. We note that $T_{nm}$ are the coefficients of the *m*-th order terms of the *n*-th order Chebyshev polynomials that obey the following three-term recurrence relation ${T_{np}=2T_{n-1,p-1}-T_{n-2,p}}$ for $p=0,\dots ,n$ (assuming $T_{np}=0$ for $p<0 \vee p>n-2$), where $T_{1k}=\delta _{1k}$ and $T_{0k}=\delta _{0k}$ being $\delta$ the Kronecker delta, whereas $T_{n0}=(-1)^{n/2}$ for *n* even and 0 for *n* odd. (These relations are easily obtained from the well-known three-term recurrence relation of Chebyshev polynomials, $T_n(x)=2xT_{n-1}-T_{n-2}$.)

Now, each $\cos ^q\theta$ term appearing in (8) can be expanded in terms of Chebyshev polynomials through the inverse formula:

9 $$\begin{aligned} \cos ^q\theta =2^{1-q}\mathop {{\mathop {\sum }\nolimits '}}\limits ^q_{\begin{array}{c} k=0\\ (q-k) \mathrm {even} \end{array}}{q\atopwithdelims ()\frac{q-k}{2}}T_k(\cos \theta ), \end{aligned}$$

where the *primed* even-order sum means that the $k=0$ term has to be halved. Since we are interested in the $P_{n0}$ coefficients appearing in (8) and only the even order powers of $\cos \theta$ have a zeroth-order term, the final expression reads

10 $$\begin{aligned} P_{n0}=\sum _{p=0}^nT_{np}\sum _{q=0}^p{p\atopwithdelims ()q}\alpha _1^{p-q}\alpha _2^qC_q. \end{aligned}$$

where

11 $$\begin{aligned} C_q=&{\left\{ \begin{array}{ll} \frac{(q)!}{2^{q}(q/2!)^2}&{} q\quad \textrm{even}\\ 0 &{}q\quad \textrm{odd} \end{array}\right. } \end{aligned}$$

is obtained for $k=0$ in (9), and recalling that for odd *q* no contribution is expected to $P_{n0}$. From the analytical expression of $P_{n0}$ originally derived here one can easily obtain the expressions of the first four $P_{n0}$ terms reported in^9^, p. 574.

## Numerical implementation

**Figure 3:**
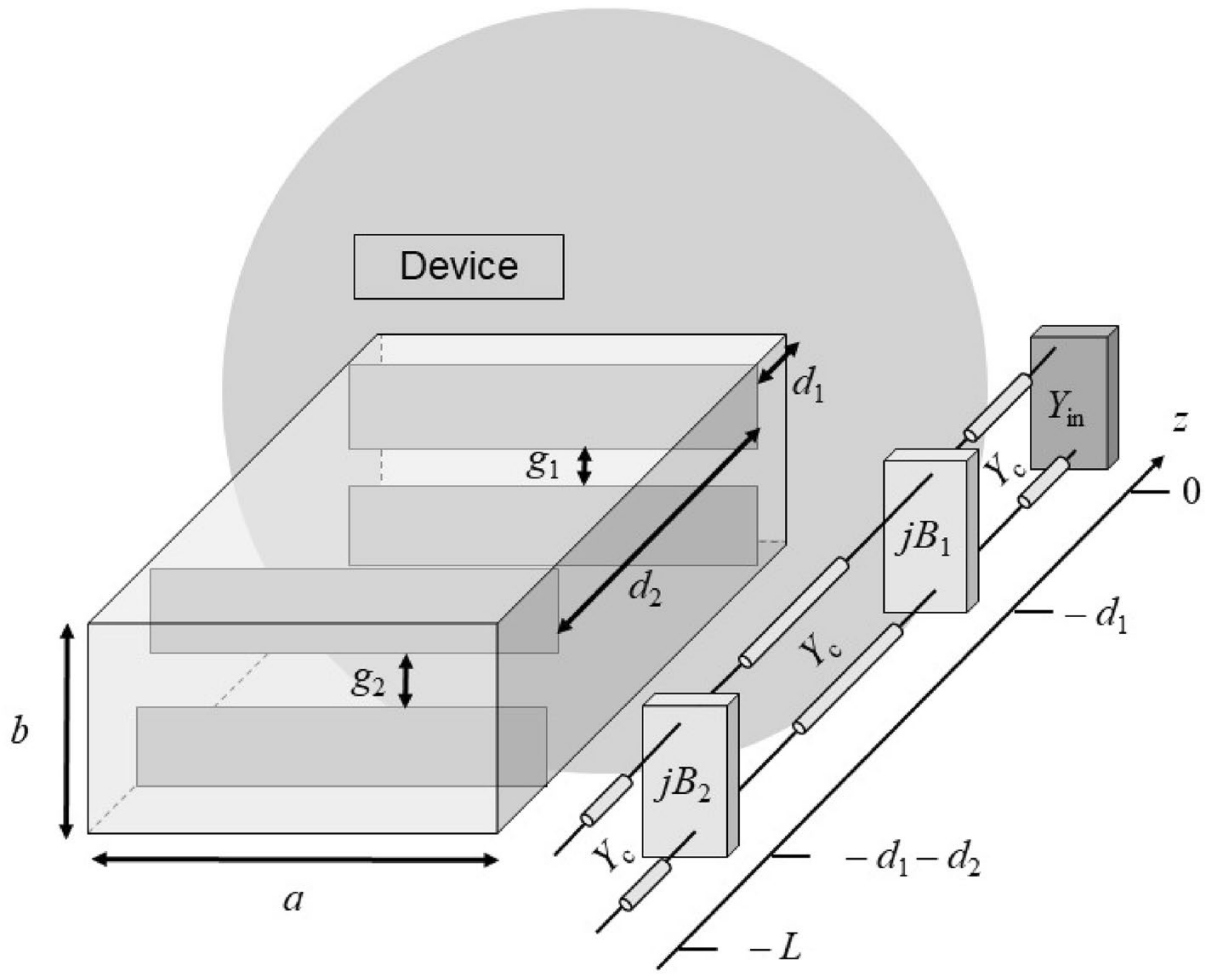
Pictorial representation of the analyzed matching structure constituted by two capacitive irises along with its equivalent transmission-line network. The excited device, in particular, is totally described by its equivalent input admittance $Y_{\textrm{in}}$ achieved through a single full-wave simulation.

Once a simple numerical representation of a matching iris has been achieved, it is possible to design a feeding network constituted by two irises capable of matching the RWG feeder of any device (see Fig. 3). As discussed in the Introduction, RWGs have often been used in order to excite different kinds of antenna and they need to be matched in order to improve the insertion-loss parameter or increase the impedance bandwidth. Whatever the excited structure is, the numerical approach proposed in this work only requires a single full-wave simulation. In particular, the entire RWG feeding section of length *L* without matching irises and with the waveguide port de-embedded up to the feeding slot has to be simulated once. Through frequency-domain full-wave solvers (here CST Microwave Studio^19^ has been used), it is then possible to extract and store the input admittance $Y_\mathrm{in}$ of the device in the desired working frequency range at the reference plane. It is worthwhile to emphasize that, by considering a single simulation to retrieve the input admittance of the entire radiating device at the source feeding slot location, we are tacitly assuming that $Y_\mathrm{in}$ does not change much when inserting the irises in the RWG feeder, due to the negligible coupling between the radiative structure and the feeding section. This approach has been validated through various analyses of practical cases, as shown in section  “Case studies”.

Therefore, the overall feeding network from the waveguide port to the radiating device, represented by $Y_\mathrm{in}$, can be described by a simple equivalent TLM. In particular, by considering a matching network constituted by two different metallic irises, the transmission line represented in Fig. 3 is obtained. As shown, the proposed matching network is constituted by an iris with a gap $g_1$ at a distance $d_1$ from the fed device, and another matching iris with a gap $g_2$ at a distance $d_2$ from the first one, thus obtaining four design parameters related to the overall matching structure. Thanks to the analytical description of the symmetric capacitive diaphragm, which has been previously introduced (valid as long as the irises are not in proximity to neglect mutual coupling^11^), it is possible to numerically describe the overall problem starting from the single $Y_\mathrm{in}$ full-wave result.

In particular, it is possible to compute the input admittance at any *z* value starting from the $Y_\mathrm{in}$ placed at $z=0$. For instance, the input admittance seen at $z=-d_1$ is:

12 $$\begin{aligned} Y_\mathrm{in}^\mathrm{d_1}= Y_\mathrm{c} \frac{Y_\mathrm{in}\cos ({k_{z0}}d_1)+jY_\mathrm{c}\sin ({k_{z0}}d_1)}{Y_\mathrm{c}\cos ({k_{z0}}d_1)+jY_\mathrm{in}\sin ({k_{z0}}d_1)} \end{aligned}$$

By considering $Y_\mathrm{in}^\mathrm{d_1}+jB_1$ as input admittance for the RWG section of length $d_2$ in (12), and iterating the process also for the last feeding section of length $L-d_1-d_2$, it is possible to numerically compute the input admittance at the waveguide port plane $Y_\mathrm{in}^{\textrm{L}}$. In such a way, the voltage input reflection coefficient is given by:

13 $$\begin{aligned} \Gamma _\mathrm{in} = \frac{Y_\mathrm{c}-Y_\mathrm{in}^{\textrm{L}}}{Y_\mathrm{c}+Y_\mathrm{in}^{\textrm{L}}} \end{aligned}$$

From Eq. (13), the input reflection coefficient can readily be evaluated for the four-iris design parameters $g_1$, $g_2$, $d_1$, and $d_2$ in a purely numerical manner. Therefore, the typical optimization (see, e.g.,^7^,^14^), which requires computationally expensive full-wave simulations of the entire structure for numerous matching irises configurations, can be replaced by the fast proposed numerical approach, which only needs a single full-wave simulation to retrieve the antenna input impedance. Through a simple numerical approach, it is indeed possible to check many different irises configuration and to select the best one in terms of the desired objective function. For matching purposes, typical optimization requests are the impedance bandwidth enlargement or the $|S_{11}|$ minimization at the working frequency $f_0$.

At this point, it should be clear that the proposed approach is rather general, effective, and flexible. Indeed, it can efficiently be applied to any matching network based on RWG discontinuities for which approximate analytical expressions exist. For instance, one can use multistage transformers that can be effective to achieve wider bandwidth or can improve $|S_{11}|$ at the working frequency. In general, by increasing the number of elements, the dimension of the parameter space increases, thus probably leading to better performance. On the other hand, some constraints on the parameters solution space can suitably be applied to avoid practical issues. For instance, for the double-iris matching network, all the configurations with very small gaps can be neglected in order to avoid computationally expensive final simulations (a very fine mesh could be needed) as well as problems in the physical implementation of the discontinuities. In the same context, all the cases for which the distances among irises or the feeding slot are too small can be neglected in order to avoid mutual-coupling and realization issues.

## Case studies

The proposed numerical approach is totally independent from the specific excited device. The latter is indeed represented only by its input admittance given by a single full-wave simulation. Therefore, there is not a specific case for which the analytical approach should not be valid. For this reason, in this Section, two different and general cases are considered in order to corroborate the method robustness: an electrically large radiating device^2,16^ and a resonant BBL^7,18^. For each case, two different application examples are discussed. As concerns the case of FPC-LWAs, a generic example taken from the available literature^20^ is considered first, in order to show the potentialities of the proposed approach. After that, the numerical description of the problem is tested for the more challenging case of a wideband FPC antenna realized through a thick PRS^16^. As concerns BBLs, the approach is almost the same: first, a stand-alone, simple, focusing device is analyzed in order to minimize the $S_{11}$ magnitude at the working frequency; second, a wireless power transfer (WPT) link is considered between two BBLs. Therefore, in the last case, the significance of this approach is further confirmed by the possibility to match a transmitting device and, in turn, maximize the transmitted power also in a near-field scenario. This is a remarkable aspect, since, to the Authors’ best knowledge, the validity of a matching technique when two devices are coupled in the radiative near-field region has not been tested in the available literature, yet.

**Figure 4:**
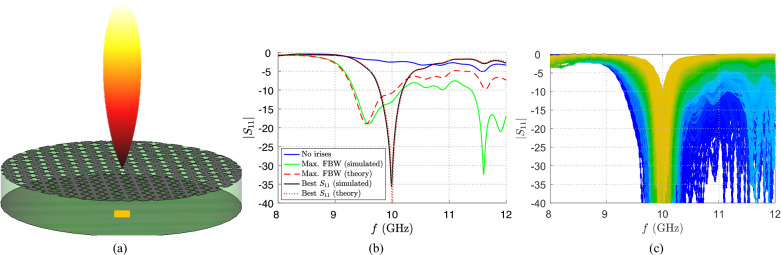
(**a**) Pictorial representation of an FPC-LWA and of its radiated pencil beam at broadside. The partially transparent green cylinder represents the dielectric substrate, its patterned upper plate the PRS and its bottom plate the ground plane. In particular, the orange rectangular structure represents the feeding area of the rectangular waveguide. (**b**) Absolute value of the $S_{11}$ scattering parameter vs. frequency *f*. The blue, green, and black solid lines represent the case of the FPC-LWA proposed by Fuscaldo^20^ when no irises are present or their design parameters are optimized for increasing the bandwidth or minimizing $|S_{11}(f_0)|$, respectively. The red dashed and dotted lines represent the $|S_{11}|$ achieved from a theoretical analysis when the FBW and the matching at $f=f_0$ are maximized, respectively. (**c**) Absolute value of the $S_{11}$ scattering parameter versus frequency *f* for different irises design parameters capable of matching the LWA shown by Fuscaldo^20^ (colors shade from blue to yellow as the matching network changes).

### Fabry-Perot cavity Leaky-Wave antenna

PRS-based FPC-LWAs consist of a grounded dielectric slab with a PRS on top and typically excited through dipole-like sources within the cavity^2,21^ (see Fig. 4a). As concerns the excitation, vertical dipoles enforce a null at broadside, thus horizontal dipoles have to be considered if a high-gain broadside pencil beam is desired. An HMD is well represented by a slot etched on the ground plane and fed with an RWG. This is also the feeder used for the first realizations of FPC antennas in the microwave range^22,23^. As concerns the PRS, various realizations exist as shown in^2^. As it is typically done in the microwave and millimeter-wave frequency ranges, we here refer to its realization through an isotropic metasurface based on metallic sub-wavelength periodic arrangements^2,3^. Such kinds of PRS are conveniently represented by a scalar imaginary impedance sheet $Z_\mathrm{s}=jX_\mathrm{s}$. This approximation holds true especially when the analysis is limited to broadside radiation as in the case studied here.

In particular, in this Subsection we consider the case of a waveguide-fed PRS-based FPC-LWA working at microwave frequencies as the one studied by Fuscaldo^20^. The proposed FPC-LWA works around $f_0 = 10$ GHz and it is realized through a half-wavelength thick FR-4 substrate (whose real part of the relative permittivity and loss tangent at 10 GHz are $\varepsilon '_r=4.3$ and $\tan \delta =0.025$, respectively) with a homogenized fishnet-like metasurface whose equivalent admittance is $X_s=50~\Omega$. The feeder is a standard RWG section of length $L=\lambda _0$, width $a=2\lambda _0/3$, and height $b=\lambda _0/3$, being $\lambda _0 = 30$ mm the vacuum operative wavelength. The waveguide feeder is coupled with the radiating device through a quasi-resonant rectangular slot (same size of the RWG cross section) on the ground plane, as shown in Fig. 3.

**Figure 5:**
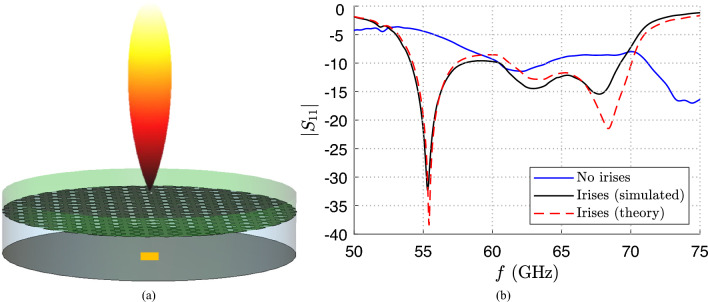
(**a**) As in Fig. 4a but with an air-filled substrate in blue and a dielectric superstate (again in green). (**b**) Absolute value of the $S_{11}$ scattering parameter vs. frequency *f*. The blue solid line represents the initial scattering parameter of the FPC-LWA proposed by Almutawa et al.^16^ when no irises are present. The red dashed line and the black solid line represent the $|S_{11}|$ achieved from a theoretical analysis and from a full-wave simulation with the optimized feeding network, respectively.

If the device is excited directly by the waveguide feeder without any matching network, the impedance matching is unsatisfactory as is manifest from the $|S_{11}|$ results (blue solid line in Fig. 4b) obtained through a full-wave implementation of the entire 3D-model of the device on CST Microwave Studio^19^. This is due to the total mismatch between the feeding structure and the radiating device. This issue, as previously discussed, can be solved by inserting two uncoupled matching capacitive irises in the waveguide section.

By applying the proposed numerical approach for $N_\mathrm{p}=21$ different values for each design parameter, it is possible to theoretically achieve the $S_{11}$ envelope versus frequency of about 200,000 different configurations in a few seconds. Among all these results, it is possible to select only the feeding networks for which the device is matched at the desired working frequency, i.e., the $S_{11}$ absolute value at the working frequency $f_0=10$ GHz is below a certain threshold set, in this case, at $-10$ dB. This result is achieved by only about 4500 configurations whose $S_{11}$ curves are reported in Fig. 4c with different colors.

Among these curves, it is possible to specifically select those that have some additional desirable features. For example, typical requests are the maximization of the operating fractional bandwidth (FBW) and the minimization of the $S_{11}$ absolute value at $f=f_0$. These two cases along with their respective design parameters are reported in Table 1, and validated with full-wave simulations to corroborate the efficiency of the proposed approach.

**Table 1:** Design parameters of the matching irises for different practical applications.

Case study	$\lambda _0$ (mm)	$d_1/\lambda _0$	$d_2/\lambda _0$	$g_1/b$	$g_2/b$
FPC-LWA with thin PRS (max bandwidth)^20^	30	0.05	0.1025	0.025	0.1
FPC-LWA with thin PRS (min $|S_{11}|$ at $f_0$)^20^	30	0.125	0.68	0.225	0.2
FPC-LWA with thick PRS^16^	5	0.225	0.155	0.075	0.1
Stand-alone BBL (TE resonance)^7^	10	0.2	0.575	0.475	0.025
WPT link between BBLs (TM resonance)^7^	10	0.075	0.68	0.1	0.1

As shown in Fig. 4b, the theoretical (red dashed and dotted lines) and the full-wave results (green and black solid lines) have an impressive correspondence for all the simulated frequencies. It is worthwhile to comment that the agreement for the case ‘Max FBW’ is not as good as that for the case ‘Best $S_{11}$’, at higher frequencies. This is due to the very small electrical dimension of *d* and $g_1$ (see Table 1) in the former case that may lead to mesh inaccuracies in the full-wave solver.

In any case, it is clear that the fractional impedance bandwidth of this device is as narrow as few percents. This, however, does not represent an issue since FPC-LWAs based on *thin* PRS as the fishnet-like metasurface usually exhibit a narrow fractional –3 dB gain bandwidth^24^. More precisely, the product between the gain peak at broadside and the –3 dB gain fractional bandwidth in PRS-based FPC-LWAs is constrained to a constant equal to $2.47/\varepsilon ^\prime _{r}$^25^. Such a constraint has been relaxed through the introduction of the so-called *thick* PRS (see Fig. 5a), discussed by Almutawa et al.^16^. Therefore, in that case, it is important to be able not to waste the theoretically available gain bandwidth with a limited impedance bandwidth, i.e., a limited working frequency range for which it results $|S_{11}|<-10$ dB.

For this purpose, the proposed numerical approach is applied to the challenging scenario of the thick PRS studied by Almutawa et al.^16^. Since such structure considers an HMD excitation, a RWG can be considered as a feeder, and thus the matching network can be designed with the same procedure outlined above.

The design of the thick PRS allows for achieving a theoretical bandwidth of the order of 15.7 GHz (from $f_1=54.25$ GHz up to $f_2 = 69.95$ GHz), which is about $25\%$ fractional bandwidth^16^. When this structure is fed with a RWG of length $L=\lambda _0$, width $a=2\lambda _0/3$, and height $b=\lambda _0/3$, being $\lambda _0=5$ mm the vacuum wavelength at the working frequency $f_0=60$ GHz, the $|S_{11}|$ reported in Fig. 5b with a blue solid line has been obtained on CST. As is shown, the desired enlarged theoretical bandwidth is not covered by the effective impedance bandwidth because the $|S_{11}|$ is lower than the $-10$ dB threshold only in the frequency range $f\simeq 60.5$–64 GHz.

**Figure 6:**
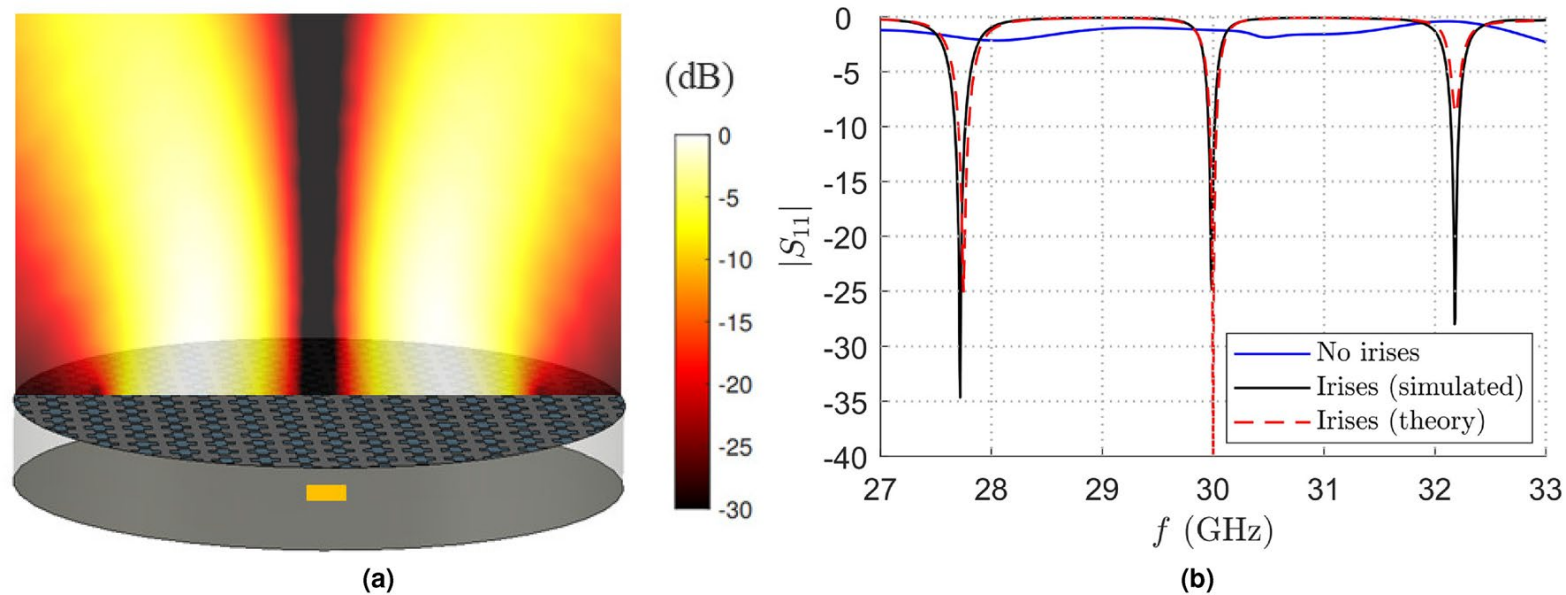
(**a**) Pictorial representation of the aperture-fed (orange rectangle) resonant BBL constituted by a metallic cavity (in grey) with an isotropic metasurface on top (patterned cylinder upper plate). Moreover, the electric field distribution is reported in a qualitative manner through a colormap in dB normalized with respect to its maximum. (**b**) Absolute value of the $S_{11}$ scattering parameter vs. frequency *f*. The blue solid line represents the initial scattering parameter of the TE-polarized BBL in^7^ when no irises are present. The red dashed line and the black solid line represent the $|S_{11}|$ achieved from a theoretical analysis and from a full-wave simulation with the optimized feeding network, respectively.

For this reason, a matching network consisting of two capacitive irises has been considered and optimized to achieve the largest impedance bandwidth. The design parameters of the irises configuration are reported in Table 1 as well; they have been obtained through a fully numerical parametric analysis testing about 12000 possible designs. The best theoretical reflection coefficient (with a small slightly not-matched frequency band) expected by the numerical approach is represented by a red dashed line in Fig. 5b. The expected envelope almost overlaps with the black solid line which describes the $|S_{11}|$ extracted by the full-wave simulation of the device (excited through the RWG with the optimized matching network). Although a very peculiar case has been considered for which the working frequency is not the central frequency of the desired bandwidth, the theoretical approach shows similar results to the simulated one that are also slightly better. Moreover, thanks to the introduction of the designed matching irises, it has been possible to obtain an impedance bandwidth that covers almost the total theoretically available gain bandwidth.

### Aperture-fed Bessel-beam launchers

Another important case study is that of a BBL (see, e.g.,^26^). Bessel-beam launchers are microwave focusing devices capable of generating Bessel beams in the radiative near-field region. In the last decades, Bessel beams gained a great interest in wireless applications (see, e.g.,^27,28^) due to their attractive properties of field focusing, limited-diffraction, and self-healing (the capability to reconstruct themselves if a scatterer obstructs its line of sight)^29^. Bessel-beam launchers are commonly distinguished between wideband (e.g.,^30–33^) and resonant devices (e.g.,^7,17,27^). The latter are particularly suited for WPT applications in the radiative near-field region^34^ where the bandwidth performance is of little concern, whereas the compactness of the device is a primary aspect^18,35–37^.

The early microwave and millimeter-wave realizations of BBLs were fed through coaxial probes inherently exhibiting a purely transverse-magnetic (TM) polarization^17,26,38^. Such a feeding technique is no longer available towards sub-millimeter frequencies. In order to overcome this issue, aperture-fed BBLs (whose pictorial representation is reported Fig. 6a) have been recently investigated by Negri et al.^7^. The Authors have shown that an excitation scheme consisting of a waveguide-fed slot (as the FPC-LWAs discussed in the previous Subsection) is capable of effectively exciting a Bessel beam with a hybrid-polarized character. Although the polarization of aperture-fed BBLs has always a hybrid character, it is possible to design the structure to resonate with the TE or the TM leaky modes. It is demonstrated that both cases achieve an excellent performance when used in a WPT scenario.

This case study thus represents an excellent example to test the matching technique proposed in this work also in a near-field scenario. As a matter of fact, capacitive irises have already been considered as matching components for aperture-fed BBLs, but their optimal design parameters have been achieved through a computationally expensive full-wave optimization of the entire structure. By considering, for example, the case of the TE resonance of the BBL in^7^, it is possible to realize a specific matching structure of the device by optimizing the irises design parameters in order to minimize the insertion loss at the working frequency $f_0=30$ GHz. As opposed to the case of thick PRS in FPC-LWAs, we are interested in minimizing the $|S_{11}(f=f_0)|$ value and we are not interested in its impedance bandwidth due to the intrinsic resonant behavior of the cavity. This aspect underlines the power and flexibility of the proposed technique. Having the chance of considering a lot of matching configurations in few seconds thanks to the totally numerical approach after a single full-wave simulation, it is indeed possible to choose the best matching structure depending on the specific application scenario of the device. For the particular case of the TE resonance shown in^7^, the best irises parameters are reported again in Table 1 and the achieved $S_{11}$ envelope is reported in Fig. 6b.

**Figure 7:**
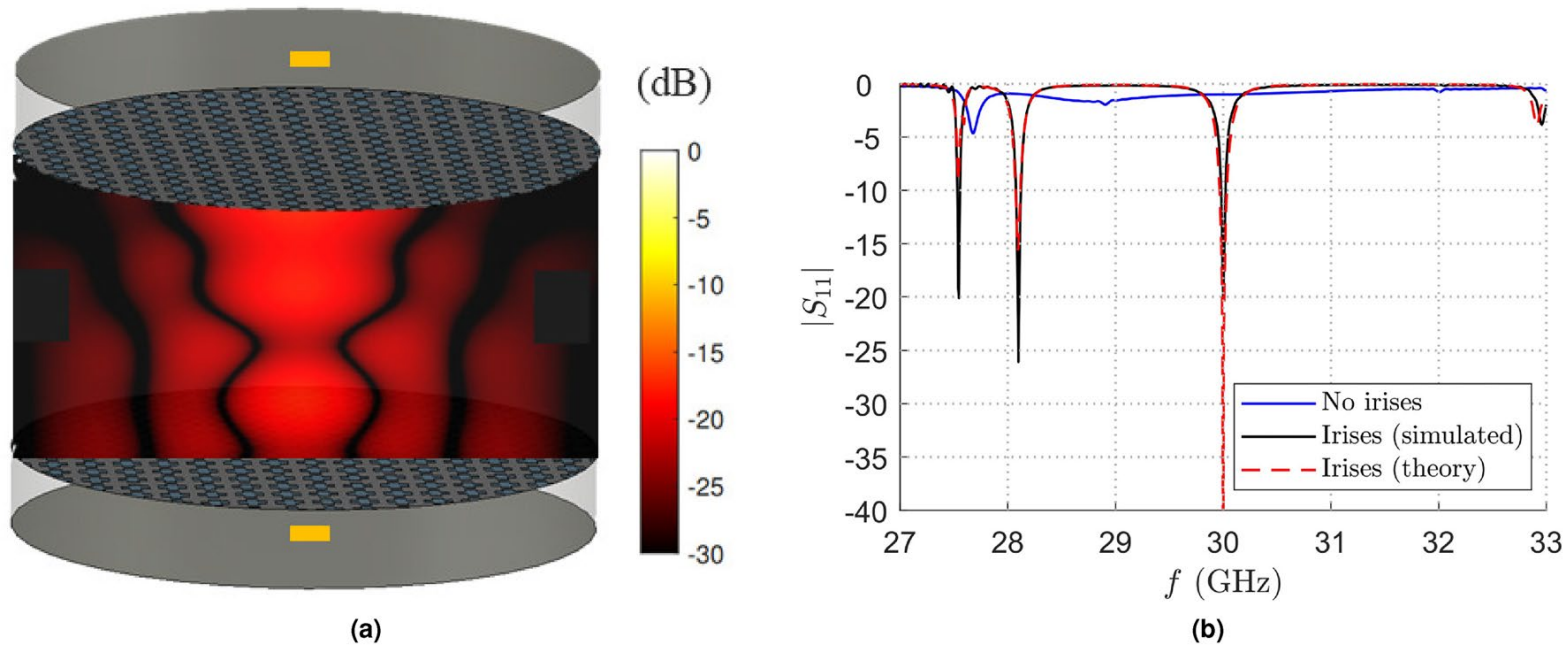
(**a**) Pictorial representation of a link between two TM-polarized aperture-fed resonant BBLs placed at a distance of 20 mm. The absolute value of the vertical component of the Poynting vector normalized with respect to its maximum is qualitatively reported through a dB color map. (**b**) Absolute value of the $S_{11}$ scattering parameter versus frequency *f*. The blue solid line represents the initial scattering parameter of the transmitting TM-polarized BBL without irises when the same device is considered on the receiving side at a distance of 20 mm^7^. In the same working scenario, the red dashed line and the black solid line have been achieved from a theoretical analysis and from a full-wave simulation with the optimized matching structure, respectively.

An even more interesting application case is given by the design of a matching network for a WPT link between two resonant BBLs coupled in the radiative near-field region (see Fig. 7a). In particular, a link between two identical resonant BBLs resonating with TM leaky modes and placed at a distance of 20 mm has been considered. As shown in Fig. 7b, the proposed approach works very well even in this challenging situation. This result is at first surprising, since it was not expected that the proximity of a load (the receiving BBL) in the radiative near-field region would have had little effect on the input admittance (and in turn the $|S_{11}|$) of the transmitting BBL. Also in this case, as for the excitation of a single resonant BBL, the best irises parameter reported in Table 1 have been chosen in order to minimize the insertion loss at the working frequency $f_0=30$ GHz, due to the resonant behavior of the link.

## Conclusion

In this paper, a classic theoretical approach capable of describing capacitive matching irises in rectangular waveguides has been reported, including also an original analytical derivation. Starting from this consolidated description, an innovative hybrid numerical/full-wave approach has been presented in order to match any device fed by a rectangular waveguide, such as planar devices whose dipole-like source is represented by a slot on the ground plane.

Although the proposed method is based on well-known electromagnetic models, its application to such diverse contexts is rarely found in the literature. The effectiveness and versatility of the proposed approach relies on its computational speed and its total independence from the specific excited devices, as demonstrated in this work by the application of the method to many different practical and challenging cases.

These features are due to the representation of the fed device only through a single full-wave simulation capable of totally describing it by its input admittance. Therefore, thanks to the analytical and theoretical approach to the problem given by a transmission-line representation, the proposed method is capable of matching any kind of device with a negligible computational burden. In such a way, the numerical matching-structure design is much faster than a full-wave optimization because the latter needs more and more computational time (due to a large number of simulations of the entire device).

## Data Availability

The datasets used and/or analysed during the current study available from the corresponding author on reasonable request.
